# Biological responses of an elite centipedegrass [*Eremochloa ophiuroides* (Munro) Hack.] cultivar (Ganbei) to carbon ion beam irradiation

**DOI:** 10.3389/fpls.2024.1433121

**Published:** 2024-09-18

**Authors:** Yuan Zhang, Haoran Wang, Yan Du, Ling Zhang, Xiaohui Li, Hailin Guo, Jianxiu Liu, Libin Zhou, Xin Xu, Jianjian Li

**Affiliations:** ^1^ The National Forestry and Grassland Administration Engineering Research Center for Germplasm Innovation and Utilization of Warm-season Turfgrasses/Jiangsu Key Laboratory for the Research and Utilization of Plant Resources, Institute of Botany, Jiangsu Province and Chinese Academy of Sciences, Nanjing Botanical Garden, Mem. Sun Yat-Sen, Nanjing, Jiangsu, China; ^2^ Biophysics Group, Biomedical Center, Institute of Modern Physics, Chinese Academy of Sciences, Lanzhou, China

**Keywords:** centipedegrass, carbon ion beam, morphological traits, physiological and biochemical characteristics, SSR analysis

## Abstract

Carbon ion beam irradiation (CIBI) is a highly efficient mutagenesis for generating mutations that can be used to expand germplasm resources and create superior new germplasm. The study investigated the effects of different doses of CIBI (50 Gy, 100 Gy, 150 Gy, 200 Gy and 300 Gy) on seed germination and seedling survival, seedling morphological and physiological traits of an elite centipedegrass cultivar Ganbei. The results showed that irradiation greater than 50 Gy cause inhibition of seed germination, and the semi-lethal dose (LD50) is around 90 Gy for CIBI treated seeds of Ganbei. A carbon ion beam-mutagenized centipedegrass population was generated from Ganbei, with irradiation dosages from 50 Gy to 200 Gy. More than ten types of phenotypic variations and novel mutants with heritable tendencies mainly including putative mutants of stolon number, length and diameter, of internode length, of leaf length and width, of leaf chlorophyll content, of stolon growth rate, of aboveground tissue dry weight, of sward height were identified. While the total sugar content of the plants from irradiated seeds showed no obvious change in all treatments as compared to the control, the crude protein content displayed significant reduction at a high-dose treatment of 200 Gy. Genetic polymorphism was detected in mutagenized centipedegrass population using SSR-PCR analysis, suggesting that CIBI caused alteration of larger fragments of the DNA sequence. As a result, a preliminary batch of mutants was screened in this study. In summary, carbon ion beam mutagenesis is an effective way for developing centipedegrass germplasm with wider variation, and treating seeds with CIBI at a dosage of ~100 Gy could be effective in centipedegrass mutation breeding.

## 1 Introduction

It is well known that practical breeding depends on genetic variation/diversity, and induction of genetic alterations and access to a range of genetic variations are critical to the success of breeding programs (Hoisington et al., 1999). Mutations are the major source of all genetic variations existing in any organism, including plants (Kharkwal, 2012). In nature, spontaneous mutations occur so rarely that they are difficult for the breeders to use in plant breeding programs. On the contrary, induced mutagenesis can potentially increase the mutation rate of plants hundreds to thousands of times beyond the natural range. The application of mutation techniques, mainly including physical mutagens and chemical agents, has successfully created a large number of genetic variations. Thereby, mutagenesis breeding has been favored by breeders and has shown remarkable success in crop improvement. It is currently a pillar of modern plant breeding along with crossing breeding and transgenic breeding (Shu et al., 2012). Due to the toxicity of chemical mutagens and their infrastructure requirements for safe use, physical mutagenesis has been increasingly favored by more and more scholars in plant breeding owing to its ubiquitous application, cost-effective and environmentally friendly trait as well as the development of a series of irradiation technologies (Bado et al., 2023).

Currently, ion beam is becoming an effective physical mutagen after its biological effects were recognized around 40 year ago (Abe et al., 2012). Carbon ion beam irradiation (CIBI) is a highly efficient mutagenesis technique with high linear energy transfer (LET) and relative biological effectiveness (RBE) in biological systems (Kraft, 2000; Feng et al., 2023). Owing to its higher LET compared to other physical mutagens, CIBI shows superiority in inducing DNA damage in a localized area, which is termed as clustered DNA damage (Goodhead, 1994; Asaithamby and Chen, 2011; Surdutovich and Solov’yov, 2014). Due to the difficulty to repair effectively and accurately, such clustered DNA damage results in the generation of free DNA fragments, which lead to the formation of chromosomal rearrangements and large deletions (Pastwa et al., 2003; Tsuruoka et al., 2008; Hirano et al., 2015). These chromosome rearrangements and deletions contribute to generation of more combinations of gene mutation sites and can potentially produce more mutants with advantageous traits (Ma et al., 2021). Hence, CIBI is expected to offer a breakthrough in the creation of new plant cultivars. Thus far, CIBI has been found widespread application in crop breeding and functional gene research due to its simplicity in implementation, high mutation rates, high survival rate, and a broad spectrum of phenotypes (Zhao et al., 2007; Becker et al., 1996). Specially, a series of new crop varieties have been generated through CIBI breeding (Ma et al., 2021; Bado et al., 2023).

Centipedegrass [*Eremochloa ophiuroides* (Munro) Hack.] is an important multipurpose grass species used as eco-grass, turf grass and forage grass that originated in China. *E. ophiuroides* is now widely distributed in East Asia, Southeast Asia, the eastern and southern United States, South America, and tropical northern and eastern Australia (Li et al., 2020). *E. ophiuroides* has highly developed stolons during growth in the field, and one of its typical features is the extremely developed prostrate growth habit. Vegetative propagation by stolons is the main means of its reproduction. It has high ornamental value and typical characteristics of higher aluminum tolerance, lower incidence of pests and diseases, and better adaptation to infertile soils as compared to other grass species (Islam and Hirata, 2005; Wang et al., 2021a). Especially, *E. ophiuroides* has been showing extraordinary performance in soil and water conservation, urban landscaping, and land surface greening in acidic soil areas, which has attracted more attention in achieving goals of health and sustainable development in China in recent decades (Xu et al., 2023). However, *E. ophiuroides* has some native defects, such as slow sward formation, low seed yields, and poor tolerance to cold and drought stresses etc., which are hindering its large-scale application in pratacultural industry. Therefore, improving these deficient traits of *E. ophiuroides* is urgent. The National Main Warm-season Turfgrass Gene Bank (NMWTGB) at Nanjing Botanical Garden, Mem. Sun Yat-Sen, China, has collected and preserved around 230 accessions of *E. ophiuroides* genetic resources (more than 40 wild accessions supplemented after 2020), including 13 cultivars registered (twonew cultivars bred and released after 2020), which are the richest and largest collections among the global *E. ophiuroides* germplasm banks and are very helpful in developing improved cultivars (Li et al., 2020). Nevertheless, the relatively narrow genetic base in *E. ophiuroides* is the main bottleneck for continued improvement of this grass species (Milla-Lewis et al., 2012; Li et al., 2020). Therefore, to enlarge interspecific variation through the creation of new germplasm is of great significance for genetic improvement of *E. ophiuroides*.

Mutation breeding based on ion beam irradiation has been playing important roles in the cultivation of new plant varieties. Generally, the mutagenic effect of radiation mutagenesis on sexually reproduced plants is performed based on M_2_ or/and M_3_ generation, while that is derived from M_1_ generation for vegetative or asexually propagated plants. Accordingly, in this study, we investigated the effects of different doses of carbon ion beam irradiation on morphological, physiological, and biochemical characteristics in the radiation-derived population of *E. ophiuroides* throughout two consecutive growing seasons. In addition, radiation induced genetic variability was further analyzed for all the radiation-derived offsprings via SSR-PCR analysis. The main purposes of this study were to determine the optimal dose for *E. ophiuroides* mutagenesis and identify mutations with desirable phenotypes for the genetic improvement of *E. ophiuroides*, and also understand the mutagenic effects of CIBI on *E. ophiuroides* so as to establish new breeding techniques and accelerate the breeding process of grass plants.

## 2 Materials and methods

### 2.1 Plant materials and irradiation treatments

The *E. ophiuroides* cultivar ‘Ganbei’ (No. E039 in NMWTGB) was used in this study. Ganbei is an elite cultivar of *E. ophiuroides* bred through systematic selection breeding and released in 2019 by the Institute of Botany, Jiangsu Province and Chinese Academy of Sciences. It is a diploid species with chromosome number of 2n = 2x = 18 and perennial growth habit (Li et al., 2020), and its genome sequence was first released in September 2021 (Wang et al., 2021a). This cultivar is a green-solon genotype with specific characteristics for lawn usage. These characteristics include emerald green leaves, well-developed stolons and root systems, fast growth and sod formation, sparse inflorescence, strong resistance to disease and pests, high tolerance to aluminum in acid soils. The turf formed by the cultivar has good uniformity and high density, with long stay-greens and low maintenance requirements. We chose dried seeds of ‘Ganbei’ (E039) with uniform size and divided them into six groups of ~500 seeds each to treat with different doses of irradiation. The irradiation was set at 40 Gy per min with dosages of 0 (Ctrl), 50, 100, 150, 200, 300 Gy. We performed the irradiation treatments of carbon ion beam under atmospheric pressure of the Heavy Ion Research Facility in Lanzhou (HIRFL) at the Institute of Modern Physics (IMP), Chinese Academy of Sciences in 2021. The irradiated seeds were then brought back to the laboratory of the Institute of Botany, Jiangsu Province and Chinese Academy of Sciences (32°07’ N, 118°73’ E) in May for further use.

### 2.2 Determination of germination rate and survival rate

The irradiated seeds were sterilized by soaking in 75% ethanol for 5 min, then 2% (v/v) sodium hypochlorite for 20 min, followed by rinsing with sterile distilled water for three times. Three replications of a half hundred seeds from each irradiation treatment were used to test the percent germination and seedling survival rate. The seeds were placed neatly in Petri dishes (diameter 9.0 cm) containing two layers of filter paper moistened with 10 mL distilled water. The Petri dishes were then placed in an illuminating incubator with a photocycle of 14h light (25°C)/10 h dark (18°C) for 14 days. The presence of ~1.0 mm length radicle was the criterion for germination. The numbers of seeds germinated were counted daily till the 14th day. On the final count day, seeds were categorized into normal and abnormal seedlings, and ungerminated seeds. Standard germination was calculated based on the percentage of the seedlings from germinated seeds. Germination Rate = (Germinated seeds/Total seeds) * 100%. In addition, ten seedlings were taken at random from each replication for measuring radicle and plumule length (mm). All seedlings of each treatment were initially planted to seedling trays and then transplanted into pots (25 cm diameter, 17 cm depth) two months later. All surviving plants were grown in a common experimental plot under the same field condition, and a NPK compound fertilizer was applied to these plants every six months with 50 grams per pot each time. All plants were irrigated with tap water once every 3 days in spring and autumn, once every 2 days in summer and once a week in winter. The seedling survival rate was calculated according to the number of seedlings and surviving seedlings.

### 2.3 Measurement of morphological traits

Both control and irradiated plantlets of *E. ophiuroides* were subjected to morphological studies during each growing season. The morphological traits including plant height, the occurrence of stolons mainly consisting of stolon number, stolon length, stolon diameter, stolon-growth rate and internode length parameters, leaf blade length and width, were determined in this study. The occurrence of spikes for each treatment was also counted. In order to ensure trait stability across successive growing seasons (generations), all plantlets of irradiation treatments and the control have been implemented with the same management measures in each growing season, including the same amount of fertilization and watering with the same time interval, and trimming the grass on the same day with the same cutting height. In addition, the managements between different growing seasons were basically consistent, i.e., the same frequency of fertilization and watering, and the same numbers of mowing. In the rapid growing period for plants each year (From July to September), in addition to increasing frequency of management measures compared to other growing periods, we also increased observation times for various morphological traits so as to determine a relatively stable period of the trait change, which could be used as the reference time for following indicator measurements. To ensure consistency in trait measurements across successive growing seasons, trait indictors were measured at the same growing time in each growing season as much as possible, and the same leaves and stolons of all plants were selected and measured with the completely consistent detecting methods.

### 2.4 Determination of above-ground biomass

For plant seedlings in a pot derived from a single seed, all stolon and leaf tissues were harvested as a sample by cutting at ground level and oven dried at 65 °C for 48 h in the laboratory. The dry weight (DW) from each sample was used to represent the AGB sampling data.

### 2.5 Determination of chlorophyll content

We used a chlorophyll meter (SPAD-502, Minolta Camera Co. Ltd, Osaka, Japan) to measure the chlorophyll content (SPAD value). A fully developed leaf from the top of the plant was chosen for recording the SPAD values. Ten leaves were selected at random for mean SPAD value per plant seedling.

### 2.6 Determination of crude protein and total sugar content

The collected aboveground part was manually cleaned and the dead or yellow leaves were removed. Five samples for each irradiation treatment were chosen for total nitrogen and total sugar analysis. The samples were washed three times by distilled water to remove dust and other residues, dried at room temperature in a shaded place for several days till complete dryness. Finally, the dried samples were ground into powder and stored in paper bags until further analyses. Total nitrogen of the powdered sample was analyzed by the micro-Kjeldahl method (Pirie, 1955), and crude protein (CP) content was determined according to AOAC (ACOC, 2004).

The total sugars were measured through phenol sulfuric acid method after extraction of leaf powder sample (Smith et al., 1983). In brief, 0.5 g of sample powder was extracted with 70% ethanol, and then 1.0 ml of the extract was transferred into the test tube. After sequentially adding 1.0 ml of 5% phenol and 5.0 ml of 98% H_2_SO_4_, the test tube loading the mixture was vortexed carefully. The mixture was then allowed to cool to room temperature and finally the absorbance of the sample was read by an Epoch Microplate Spectrophotometer (BioTek Instruments, Inc. USA) at λ = 490 nm. The results were expressed as mg·g^-1^DW.

### 2.7 PCR analysis of genetic variation among irradiation-derived offspring

Genomic DNA was extracted from the young leaves of each plant using FlaPure Plant DNA Extraction Kit (DE711-50, Beijing Genes and Biotech). The concentration of DNA was determined by an Epoch Microplate Spectrophotometer (BioTek Instruments, Inc. USA). All of the DNA samples were diluted to a final working concentration of 30 ng/uL.

Eleven*E. Ophiuroides* simple sequence repeat (SSR) primer sets previously developed by our laboratory (Li et al., 2018) were used for testing their suitability. PCR analysis confirmed that all eleven primer pairs (**Table 1**) could amplify clear, reproducible and polymorphic bands at one or more loci in *E. ophiuroides*, so all primer pairs were further chosen to analyze the polymorphism in all irradiation-derived offspring. PCR amplification was implemented in a 10 μL reaction mixture containing 1.5 μL genomic DNA (30 ng/μL), 5μL of 2 x Taq Master Mix (AG11009, Accurate Biology), 1 μL of each primer (10 μM), and 2.5 μL of ddH_2_O. The conditions for PCR amplification comprised an initial denaturing step (95°C/3 min) followed by ten cycles of 94°C/30 s, 58°C/30 s, and 72°C/30 s, and then 25 cycles of 94°C/30 s, 55°C/30 s, and 72°C/30 s, and finally by an extension at 72°C for 10 min. The PCR products were subjected to nondenaturing polyacrylamide gel electrophoresis (PAGE) in 8.0% polyacrylamide gelsalong with 100 bp ladder under the constant supply of 120 V for 45 min. After electrophoresis, the gel was visualized by 0.1% silver nitrate staining and documented by gel documentation system (Shanhai Peiqing Science & Technology Co., Ltd, JS-mini10).

**Table 1:** SSR primer information used for PCR analysis.

No.	Marker Name	Forward sequence (5’-3’)	Reverse sequence (5’-3’)
1	TJIB.Eo_010	GGTGCTCAGTTGCAGCATAA	CGTCATACAACCGGAGGTG
2	TJIB.Eo_017	AGGGCTAGAATTAGGAGGCG	GCGTGAACGCTCACTCACT
3	TJIB.Eo_054	CTTAGCCACCACCACATCCT	GTGACCTCTAGCCATCGGAG
4	TJIB.Eo_055	GCTCGTGTGGACTACCAACA	TCCTCCTCTTCTCCTTGCTG
5	TJIB.Eo_061	TCAGCAGTTGTGCTGGAATC	CCATGGGAGTGATGATGATG
6	TJIB.Eo_065	TGAGAGAACCCTCATAACACAGA	GGAAAGGCTGTCTATGCTGC
7	TJIB.Eo_068	ATGTGAACGCTTTCCTCTCG	AGACGATCAACGCAACAACA
8	TJIB.Eo_074	CTTTGAGAGGGCGCTTATTG	TGACCTTGAGTACGTGCTGG
9	TJIB.Eo_080	AAGCGCCTTCTCCTCTAACC	ATTACTCGGAGGGTCCGTTT
10	TJIB.Eo_091	CACACTTCCGAGGTGGACTT	AGGAGTGCCCAAATCACAAG
11	TJIB.Eo_092	CTGGCATCTCTTCTGGCAC	GAGGAGGAGGAGGAGGACAG

### 2.8 Statistical analysis

The data obtained from germination experiment and biochemical determination of crude protein and total sugar contents were subjected to one-way analysis of variance (ANOVA) and Tukey’s honestly significant difference (HSD) test at the P < 0.05 level to determine the significant differences between irradiation treatments. These statistical analyses were carried out by using SPSS software (Version 23.0, SPSS Inc., USA). For the other data in the study, the box plots were created to compare the differences among different irradiation treatments. The median, 10^th^, 25^th^, 75^th^, and 90^th^ percentiles were plotted as vertical boxes with error bars in the box plot. The figures were all created with GraphPad Prism 9 software.

## 3 Results

### 3.1 Effects of CIBI on seed germination and seedling survival of *E. ophiuroides*

**Figure 1:**
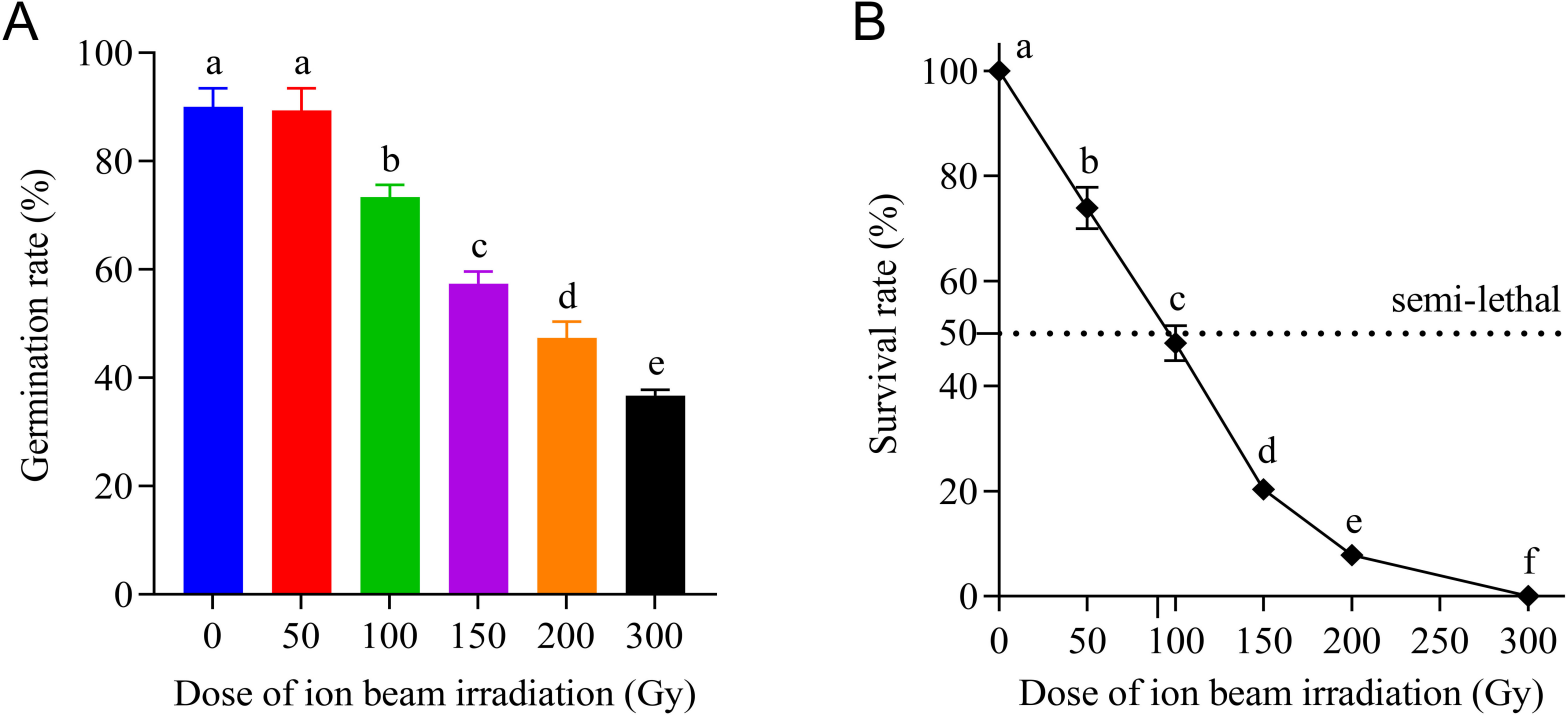
Effects of CIBI on seed germination rate and survival rate of *E*. *ophiuroides* seedlings. Means with different letters are significantly different between treatments by the Tukey’s HSD test (p<0.05). Error bars indicate the mean ± standard deviation. **(A)** The seed germination rates under different irradiation doses. **(B)** The survival rate of seedlings developed from seeds irradiated with different doses. One hundred and fifty seeds in three replications were taken from each treatment for counting percent germination.

The number of germinated seeds and the length of radicle and plumule of *E. ophiuroides* were measured over time in each irradiation dose regime. Final germination rate was unaffected by CIBI under 50 Gy, but declined rapidly as the irradiation dose increased to 100 Gyor higher. As shown in **Figure 1**, the germination rate of 89.3% at 50 Gy was basically equivalent to that of 90.0% in the control. However, when the irradiation dose exceeded 50 Gy, the germination rate decreased significantly from 73.3% at 100 Gy to 36.7% at 300 Gy, with a decreased range of 16.7-53.3%.

CIBIcaused a significant decrease in survival rate of transplanted seedlings from germinated seeds, and the decrease in magnitude of survival rate increased with the increasing dose (**Figure 1B**). The survival rates of 50, 100, 150 and 200 Gy were 73.9%, 48.2%, 20.3% and 7.9%, respectively, exhibiting an approximately linear downward trend. Especially compared to the control with survival rate of 100%, all seedlings from 300 Gy treatment died after transplanting, indicating that the dose of 300 Gy can be considered as total lethal dose for *E. ophiuroides* seed irradiation. Correspondingly, the semi-lethal dose (LD50) should be about 90 Gy for CIBI treated seeds of *E. ophiuroides*, which is crucial for determining the optimal dose for ion beam based mutagenic breeding of grass plants.

**Figure 2:**
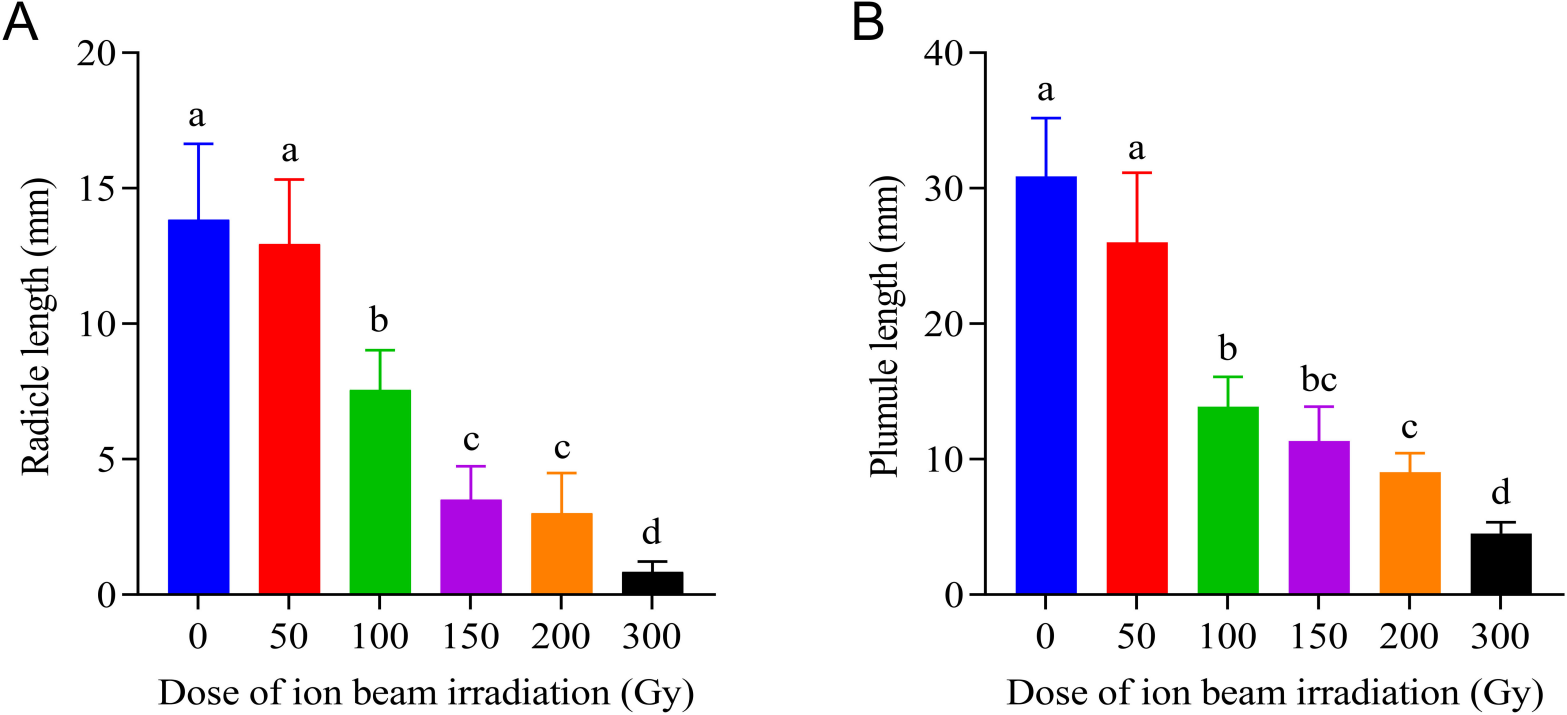
Effects of CIBI on radicle and plumule elongation in germinating seeds of *E*. *ophiuroides*. Means with different letters are significantly different between treatments by the Tukey’s HSD test (p<0.05). **(A)** The radicle lengthunder different irradiation doses. **(B)** Theplumule lengthunder different irradiation doses. 30 seedlings in three replications were taken from each treatment for measuring radicle and plumule length.

The elongation of both radicle and plumule was obviously inhibited by irradiation treatments with doses greater than 50 Gy, and the inhibitory effect increased with increasing doses of CIBI administered on the seeds. As shown in **Figure 2**, although 50 Gy treatment had no apparent influence on both radicle and plumule lengths, the higher dose treatments resulted in a significant decrease of radicle and plumule length. The radicle length decreased from 11.8mm in 50 Gy to 3.8 mm in 200 Gy with a decreasing range between 12.0% and 71.5% compared with the control. Similarly, the plumule length decreased by 15.8% in 50 Gy treatments and 69.6% in 200 Gy treatments, and the decreasing degrees increased with increasing doses of irradiation. Under 300 Gy treatment, both radicle and plumule showed almost no growth, with radicle length being less than 0.83 mm and plumule length being less than 4.5 mm which were much shorter than that in the control (radicle length 13.4 mm and plumule length 30.9 mm, respectively).

### 3.2 Morphological and physiological responses

#### 3.2.1 Statistics of visual morphological traits mutants

**Figure 3:**
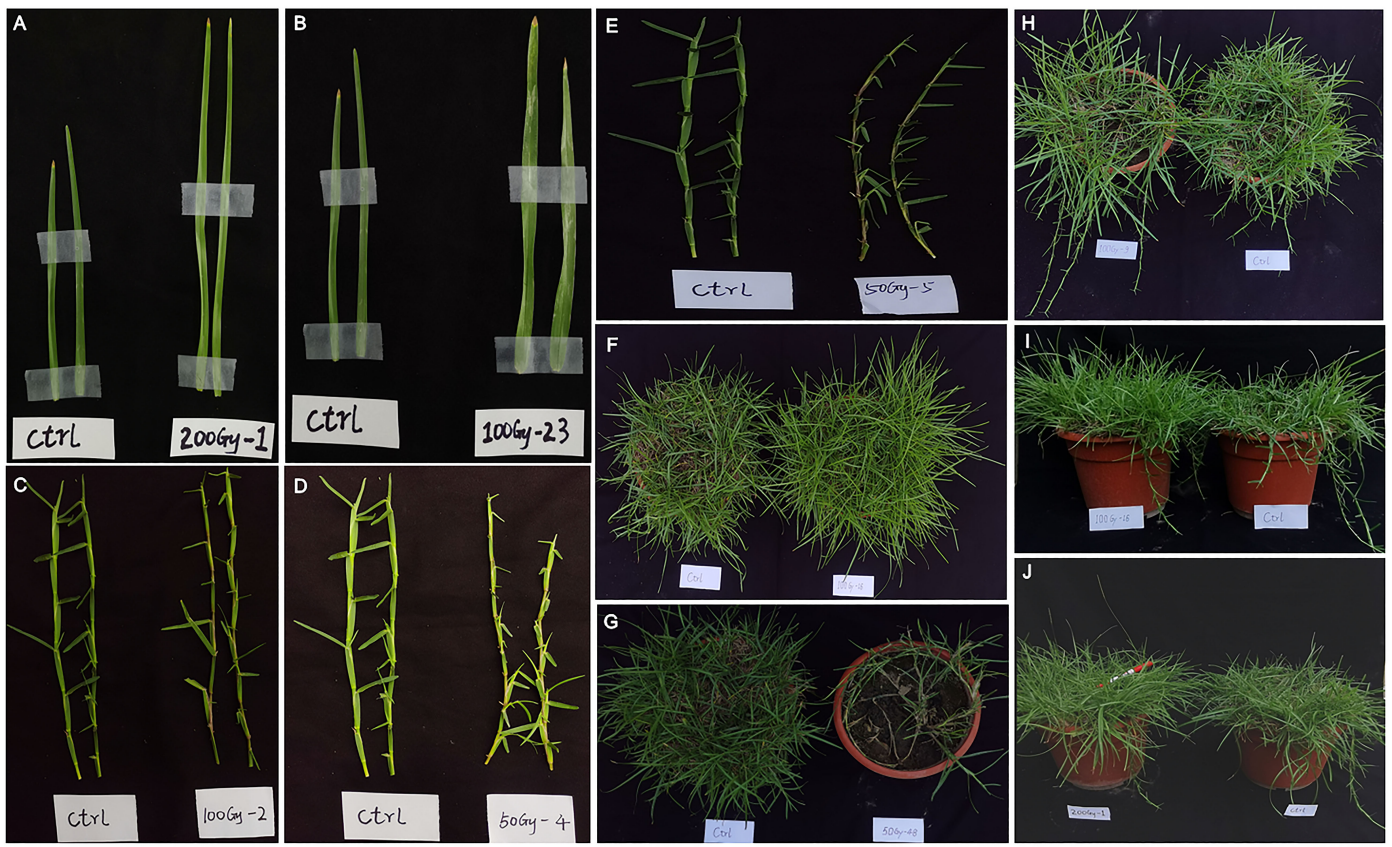
Phenotypic alterations of *E*. *ophiuroides* induced by CIBI. **(A)** Elongated blade mutant. **(B)** Widened blade mutant. **(C)** Reddened stolon mutant. **(D)** Shortened internode mutant. **(E)** Stolon thinning and reddening mutant. **(F)** Fast lawn-forming mutant. **(G)** Reduced aboveground biomass or poor lawn formation mutant. **(H)** Mutant with a rapid differentiation and number increase of stolons. **(I)** Increased sward height mutant. **(J)** Early inflorescence occurrence mutant. The control is on the left side for each of the panels **(A, G)**, and is on the right side for each of the figures of **(H, J)**.

Compared to the control, delayed germination occurred in all CIBI treated seeds and the plants from irradiated seeds grew more slowly during the seedling stage. During the later growth stages, a series of mutants with obvious phenotypic alterations were identified in the offspring of the irradiation treatments. The most noticeable variations for phenotypic traits observed during growth stage were altered blades and stolons, mainly including blades with increased length (**Figure 3A**), blades with increase width (**Figure 3B**), reddened stolons (**Figure 3C**), slotons with shortened internodes (**Figure 3D**), stolon with reduced diameter and reddened in color (**Figure 3E**). In terms of lawn formation speed, synchronously increased numbers of leaf blades and stolons, which determines the speed of lawn formation, was identified in quite a few offspring (**Figure 3F**); and extremely poor lawn formation or the failure of lawn formation was also observed (**Figure 3G**). With regard to grass expansion ability, enhanced expansion ability manifested as rapid differentiation and number increase of stolons was detected (**Figure 3H**). In addition, variation in height of sward was observed, and increased sward height occurred in some offspring of the irradiation treatments (**Figure 3I**). Specially, early inflorescence occurrence was found in a small portion of offspring (**Figure 3J**), which is important for *E. ophiuroides* selection and breeding related to seed production traits.

#### 3.2.2 Effects of CIBI on the occurrence and growth characteristics of stolons

**Figure 4:**
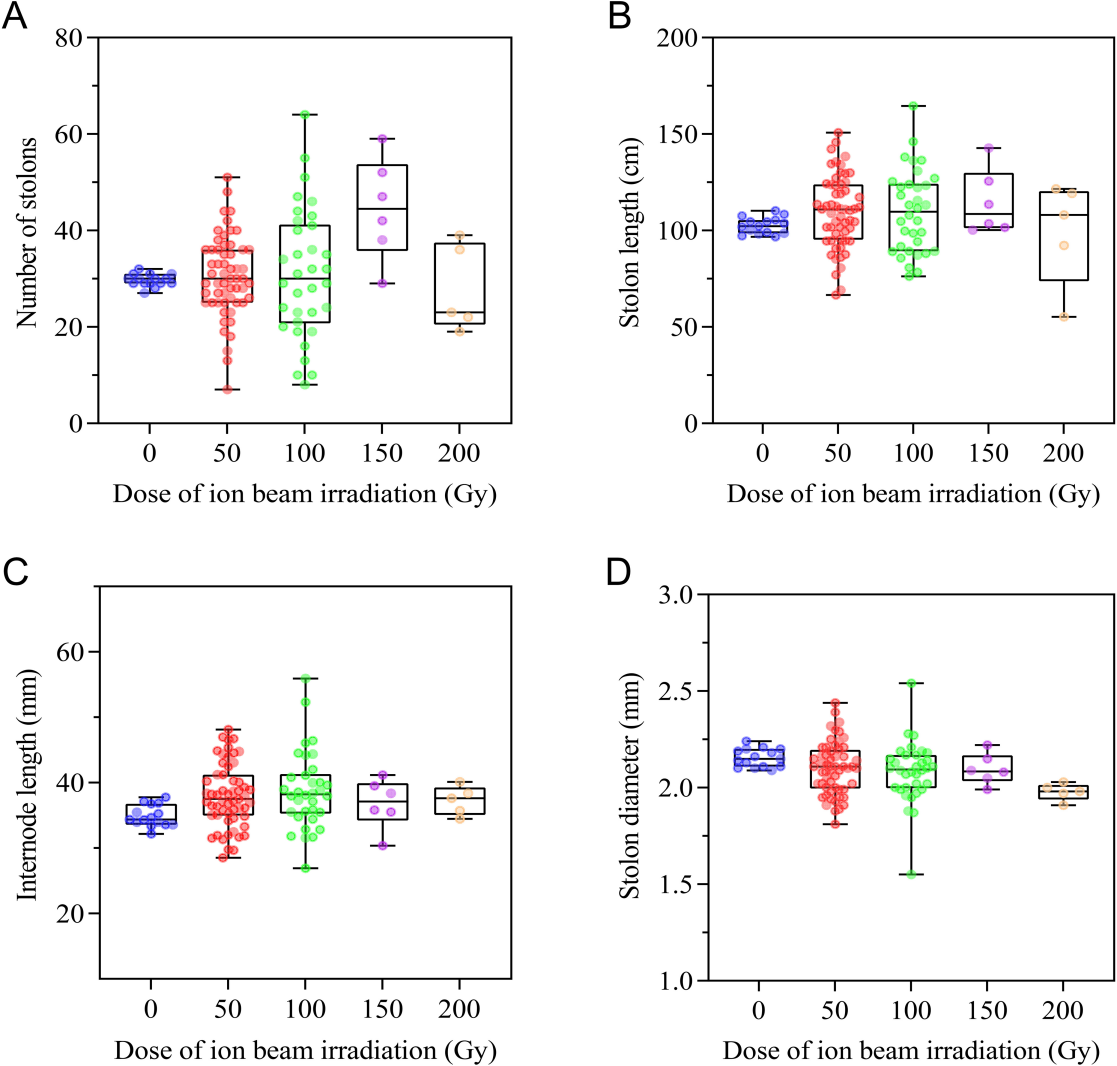
Effect of CIBI on stolon occurrence and growth characteristics in *E*. *ophiuroides*. **(A)** Number of stolons generated. **(B)** Stolon length. **(C)** Internode length. **(D)** Stolon diameter.The presence of ~5.0 cm length stolon is the criterion for counting stolon number. The first three stolons emerging from a plant were used for determining stolon length, and the length of 3-month-old stolon was measured with a tape measure from the base to tip of the stolon. Internode length and stolon diameter were measured using a ruler or vernier caliper at the position between the third and fourth nodes of the stolon, and five replicates at least were implemented for each index measurement.

Changes in occurrence and growth characteristics of stolons response to CIBI are shown in **Figure 4**. The irradiation treatments resulted in a wider variation in the occurrence of stolons in offspring of the irradiated *E. ophiuroides*, with stolon number ranging from 7 to 64 compared to that of the control (27 to 32). Of all the offspring derived from irradiation treatments, 38.6%, 41.2%, 83.3% and 40.0% population in 50Gy, 100 Gy, 150 Gy and 200 Gy treatments respectively showed greater number of stolons than the control, while 29.8%, 38.2%, 0% and 60.0% population in corresponding treatments displayed a smaller number of stolons than the control (**Figure 4A**; **Supplementary Table S1**). For stolon length and internode length, generally CIBI caused production of longer stolon length and internode length in most offspring. Compared to the control, around half of offspring in each of irradiated treatments showed a significant increase in stolon length except for 200 Gy treatment (40.0%), with 52.6% in 50Gy, 50.0% in 100 Gy and 50.0% in 150 Gy; while less than or approximately equal to one-third of offspring in each treatment, except for 200 Gy being 40%, shortened significantly their stolon length with 26.3% in 50 Gy, 32.4% in 100 Gy and 0% in 150 Gy (**Figure 4B**; **Supplementary Table S1**). Similarly, about half population of each of irradiated treatments had increased internode length except for 200 Gy treatment (40.0%), with 49.1% in 50Gy, 58.8% in 100 Gy and 50.0% in 150 Gy; less than 20% population in each treatment had significantly shortened internode length, with 0% in both 50 Gy and 200 Gy, 11.8% in 100 Gy and 16.7% in 150 Gy (**Figure 4C**; **Supplementary Table S1**). Contrary to the change in stolon length and internode length, irradiation gave rise to a generally decreasing trend of stolon diameter in most offspring of all treatments. 43.9% population in 50 Gy, 44.1% in 100 Gy, 50% in 150 Gy and 100% in 200 Gy exhibited a significant decrease in stolon diameter, while 14.0% population in 50 Gy, 8.8% in 100 Gy, and 0% in both 150 Gy and 200 Gy had significantly thickened stolon diameter (**Figure 4D**; **Supplementary Table S1**).

#### 3.2.3 Effects of CIBI on chlorophyll content and morphological characteristics of leaf blades

**Figure 5:**
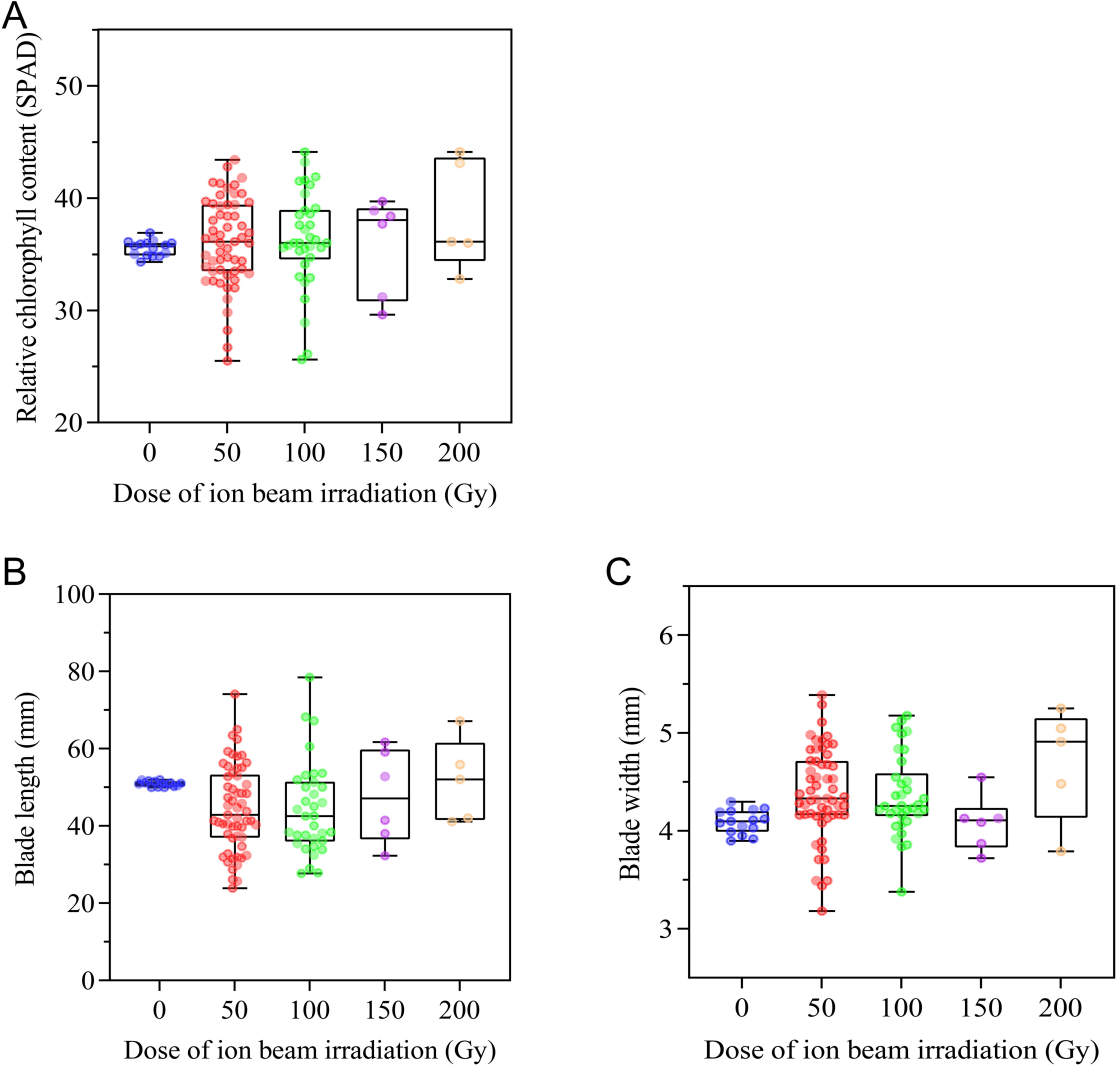
Effect of CIBI on chlorophyll content and morphological characteristics of leaf blades in *E*. *ophiuroides*. **(A)** Relative chlorophyll content. **(B)** Blade length. **(C)** Blade width. The 3rd leaf from tip was used for measuring SPAD value, blade length and width. 10 leaves were taken for each index measurement.

As an important trait related to turf aesthetics, leaf blade morphological characteristics and chlorophyll content were measured. CIBI brought about a wide range of variations in chlorophyll content, leaf blade length and width (**Figure 5**). Compared to the control with relative chlorophyll content ranging from 34.3 to 36.9, offspring from all irradiation treatments showed greater variations in relative chlorophyll content, with 50 Gy treatment ranging from 25.5 to 43.4, 100Gy ranging from 25.6 to 44.1,150 Gy ranging from 29.6 to 39.7,200 Gy ranging from 32.8 to 44.1; 31.6%, 23.5%, 33.3% and 20.0% population in 50Gy, 100 Gy, 150 Gy and 200Gy treatments had obviously decreased relative chlorophyll content, respectively; 40.4%, 41.2%, 66.7% and 40.0% population in corresponding 50 Gy, 100 Gy, 150 Gy and 200 Gy treatments showed significantly increased relative chlorophyll content (**Figure 5A**; **Supplementary Table S1**). In terms of leaf morphological characteristics, a wider range of blade length in all treatments, with 50 Gy, 100 Gy, 150 Gy and 200 Gy respectively ranging between 23.9 and 74.1 mm, between 27.7 and 78.5 mm, between 32.3 and 61.7 mm, between 41.1 and 67.2mm, relative to the control (ranging between50.0 and 52.0 mm) was found but the median values of blade length in all treatments were lower than that of the control (**Figure 5B**; **Supplementary Table S1**). CIBI also caused a wider range of blade width in offspring of each of irradiated treatments similar to that of blade length, however, the median values of blade width in all treatments were higher than that of the control (**Figure 5C**; **Supplementary Table S1**).

#### 3.2.4 Effects of CIBI on production potential

**Figure 6:**
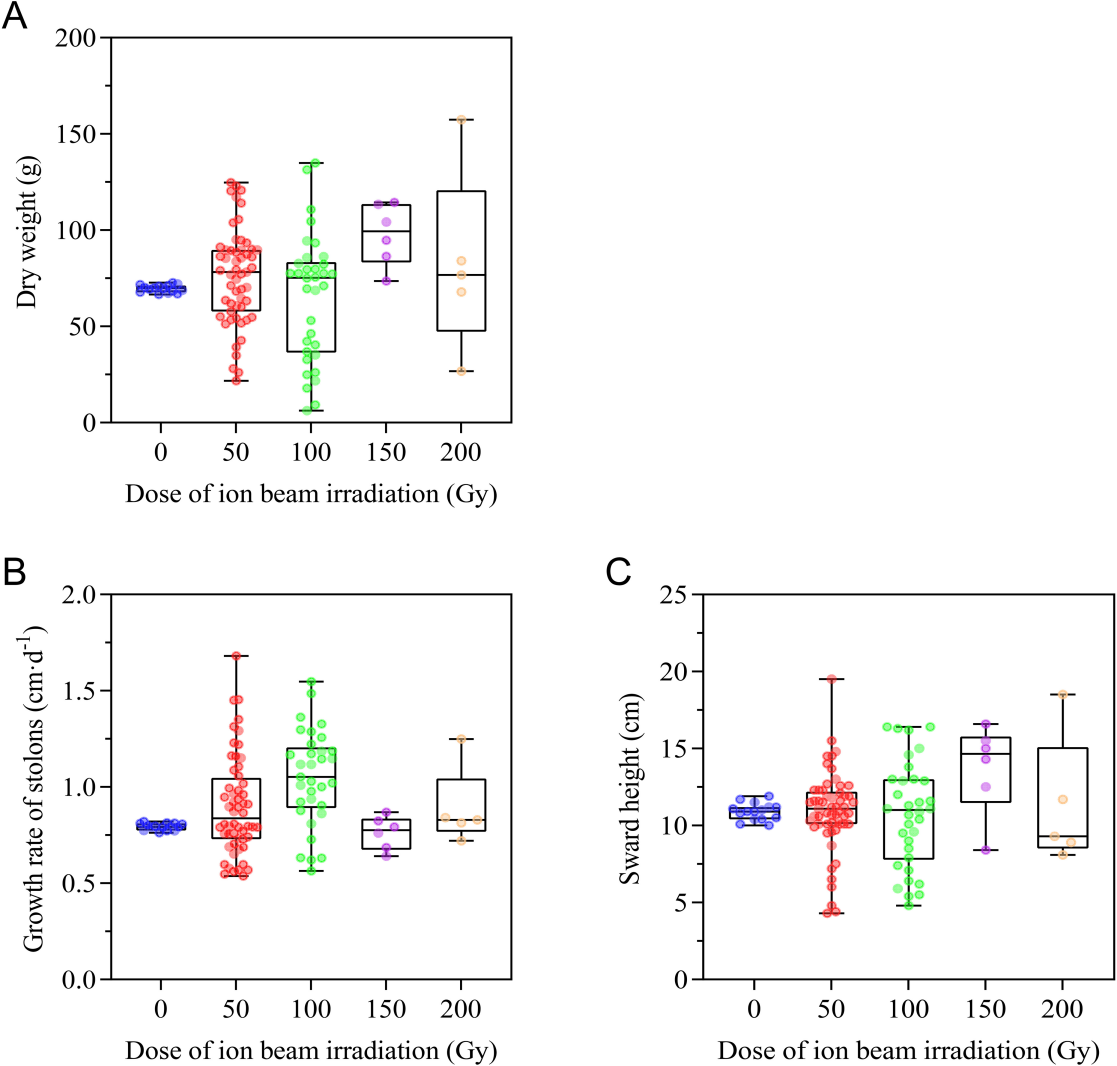
Effect of CIBI on production potential of *E*. *ophiuroides*. **(A)** Above biomass or Dry weight. **(B)** Growth rate of stolons. **(C)** Swardheight. All aboveground tissues above 5 cm of the turfgrass plug were cut for assessing above biomass before the plant went dormant or turned yellow in winter each year. Growth ratewas determined by calculating the elongation of a stolon per unit of growing time with at least five replicates for each plant. Sward height was measured at five different spots from the base level to the highest point of the lawn.

In order to assess the production potential of *E. ophiuroides*, aboveground biomass, growth rate of stolons, and sward height were measured. As shown in **Figure 6A**, CIBI treatment resulted in great variation in total aboveground biomass (dry weight). Relative to dry weight of the control ranging from 66.6 to 72.7 g, the dry weight of offspring in 100 Gy treatment showed the widest range within 6.2-134.9g, followed by 50 Gy treatment with the range within 21.8-124.7g, 200 Gy treatment within 26.7-157.4g. Although 150 Gy treatment had a relatively narrow scope relative to the other three treatments, all population of the treatment showed obviously increased dry weight compared to the control (**Figure 6A**; **Supplementary Table S1**).

Similar to aboveground biomass, both growth rate of stolons and sward height exhibited a wider range of variation among populations of all irradiated treatments compared to that of the control. For both growth rate of stolons and sward height, 50 Gy treatment had the widest range of variation, followed by100 Gy, 200 Gy and 150 Gy treatment. Compared to the control, the median values of growth rate of stolons were higher for all the irradiated treatments except for 150 Gy treatment, but the median values of sward height was highest under the 150 Gy treatment (**Figures 6B, C**; **Supplementary Table S1**).

#### 3.2.5 Effects of CIBI on crude protein content and total sugar content

**Figure 7:**
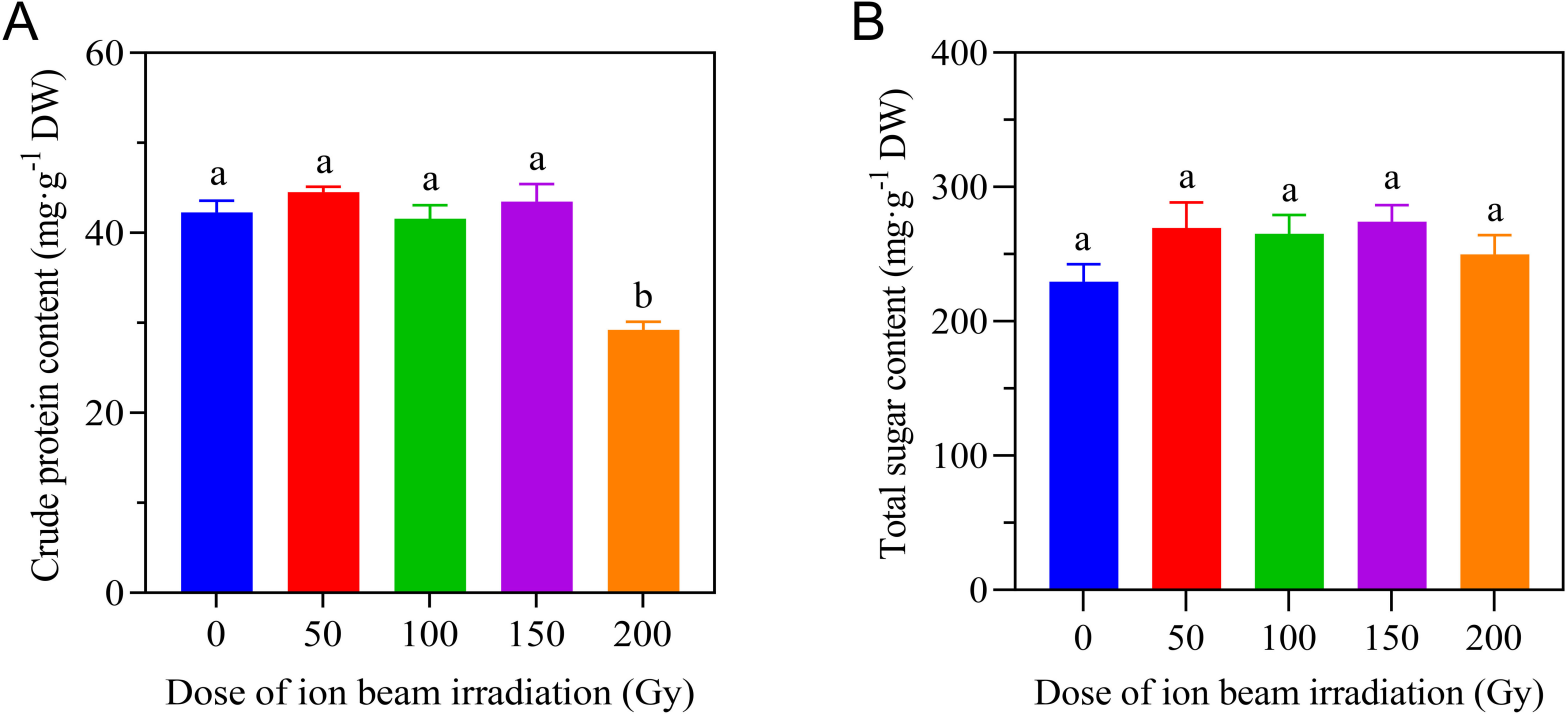
Effect of CIBI on crude protein content and total sugar content of *E*. *ophiuroides*. Means with different letters are significantly differentbetween treatments by the Tukey’s HSD test (p<0.05). **(A)** Crude protein content. **(B)** Total sugar content.Five samples were randomly selected from the collected above tissues for both crude protein and total sugar assessment.

To ascertain the effect of CIBI on the main nutritional status of the aboveground parts of *E. ophiuroides*, crude protein content and total sugar content were further tested. Compared to the control, CIBI caused a significant reduction of crude protein content only in 200 Gy treatment, while the other irradiation treatments maintained a stable level of crude protein content that was comparable to the control. As for total sugar content, no significant difference was found between each of all irradiation treatments and the control (**Figure 7**).

### 3.3 PCR analyses between the wild-type plant and irradiation-derived populations

**Figure 8:**
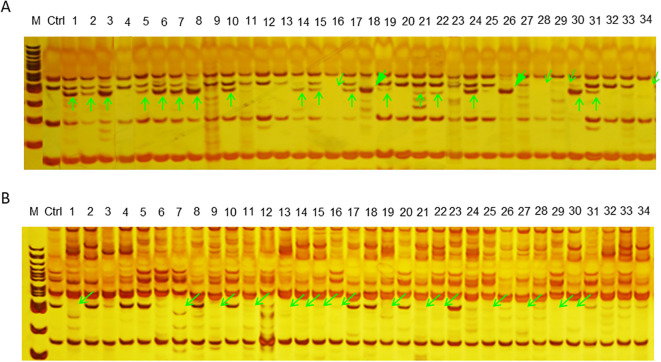
Examples of PCR analysis to identify variations between wide-type plant and the mutagenized population based on SSR markers. *M* 100 bp DNA ladder, *Ctrl* the control or wild-type plant, *1–34* the mutagenized population of *E*. *ophiuroides*. **(A)** PAGE visualization of PCR products amplified from SSR marker TJIB.Eo_068. **(B)** PAGE visualization of PCR products amplified from SSR marker TJIB.Eo_092. Different PCR amplification patterns are shown in green arrows, with vertical arrow indicating the increased bands, slanted hollow arrow indicating the missing bands, and slanted solid arrow indicating the bands with altered fragment size.

PCR amplification was carried out to compare the wild-type plants (Ctrl/E039) with the offspring derived from CIBI treated seeds using 11 SSR prime pairs. Although DNA variation between wild-type and the putative mutants was not identified by three SSR markers (TJIB.Eo_055, TJIB.Eo_05580 and TJIB.Eo_091), clear polymorphisms between them were detected by the other eight markers (**Figure 8**; **Supplementary Table S2**). Specially, a considerable number of amplified band differences were detected by markers TJIB.Eo_065 and TJIB.Eo_091. The generated polymorphisms were mainly reflected in three changes: increased number of amplified fragments with a frequency of 5.02%, reduced number of the fragments with a frequency of 5.39%, and a change in fragment size with frequency of1.59%. The polymorphism rate induced by CIBI at 100 Gy and 200Gy treats was higher than at other doses of irradiation treatments.

## 4 Discussion

Heavy-ion beam irradiation is receiving increasing attention in the field of plant breeding due to its ability to provide a broader mutation spectrum, higher mutation rates, less damage to plant material, and especially its advantage in producing a greater number of phenotypic mutants at lower radiation doses compared to other physical mutagenesis (Ren et al., 2023; Guo et al., 2023). However, like other radiation-induced mutation breeding techniques, the selection of the optimal radiation dose is crucial for heavy-ion mutagenesis. The basic principle for selecting the optimal dosage is to acquire a high mutation frequency with more beneficial mutants and obtain sufficient viable materials (like seeds). In general, the dose causing 50% lethality of plant material is chosen as the optimal radiation dose and is referred to as the median lethal dose (LD50) (Muhammad et al., 2021). The LD50 value may vary depending on the radiation source and the materials being irradiated. Although both germination rate and survival rate decreased sharply with an increasing of the irradiation dose in the present study, the LD50 of around 200 Gy based on germination rate is unrealistic because of the very low survival rate of seedlings. Therefore, the LD50 of ~90 Gy derived from survival rate is more suitable for carbon ion beam-induced mutagenesis of *E. ophiuroides.* Moreover, taking into account the occurrence of higher phenotypic variation at 100 Gy, a slightly higher dose than survival rate-based LD50 might be a preferable choice for CIBI in *E. ophiuroides.*

A series of phenotype mutants were identified in this study, including increased blade length mutants, increased blade width mutants, reddened stolon mutants, shortened internodes of stolon mutants, reduced diameters of stolon mutants, both fast lawn formation and poor lawn formation mutants, enhanced expansion speed mutants, increased sward height mutants, and early occurred inflorescence mutants. These mutants exhibited a relatively good heritability and stability in the second growing season (**Supplementary Figures S1**–**S4**), such heritable tendencies could be mainly attributed to asexual reproductive strategy of *E. ophiuroides*. Although several improved varieties of *E. ophiuroides*, like AU Centennial and TifBlair, were developed through gamma radiation mutagenesis, there has been no large-scale development of mutants in *E. ophiuroides* so far. Therefore, the mutants generated in this study are valuable germplasm resources for *E. ophiuroides* breeding and variety improvement. In addition, these mutants could be good experimental materials for clarifying mechanisms of plant growth and flower development in *E. ophiuroides* and the similar turf plants.

Visual turf quality is directly related to the color of the lawn, which is dependent primarily upon the level of pigment content in turfgrass blades. As the main factor determining the color of plant leaf blades, chlorophyll is the key pigment in photosynthesis and also important biochemical indictor for detecting the degree of blade variation in leaf color. Chlorophyll-related leaf mutations were obtained in many plant species by radiation mutagenesis, such as *Cymbidium* (Kim et al., 2020), soybean (Wang et al., 2021b), sweet pepper (Honda et al., 2006). It seems that radiation mutagenesis had a high probability of generating chlorophyll-related mutants (Sakowska et al., 2018; Arase et al., 2011; Wang et al., 2021b). In our study, both chlorophyll increase and decrease mutants were found in offspring of CIBI treated *E. ophiuroides*, and the incidence of chlorophyll increase mutation is higher than that of chlorophyll decrease mutation. Specifically, we found a higher incidence of chlorophyll mutants with increased doses of CIBI treatments in *E. ophiuroides*. The consistent findings were also reported in cowpea (Singh et al., 2013) and urdbean (Goyal and Khan, 2010). These results demonstrate that radiation mutagenesis has certain advantages for induction of chlorophyll-related mutants in plants.

Besides used as a turfgrass in lawns, *E. ophiuroides* is also used in permanent pastures for its capability of withstanding heavy grazing (Cook et al., 2005; Heuzé et al., 2016). However, as a forage use, the nutritional level of a grass species should be particularly noteworthy. In general, crude protein content and total sugar content are used for nutritional assessment of forage quality. In this study, there was no significant change in crude protein and total sugar contents for CIBI-derived aboveground tissues, except for the plant tissues of 200 Gy treatment with a decrease in crude protein content. This seems to indicate that ion beam irradiation did not cause change in nutritional quality of *E. ophiuroides*. But for their stability in heritable tendency, further continuous testing based on multiple growth seasons is to be necessary.

Whether used as turfgrass or forage grass, *E. ophiuroides* inevitably encounters various environmental stresses during its growth and development process. As sessile plants, they have to adapt a wide range of environmental conditions or tolerant to a diverse array of abiotic stressors (Singh et al., 2018). In plants, a common response to adverse stresses is the accumulation of sugars. Sugars functions as a nutrient, regulator as well as a signaling molecule of metabolism, growth, stress responses and development from embryogenesis to senescence (Rolland et al., 2006; O’Hara et al., 2013; Smeekens and Hellmann, 2014; Sami et al., 2016). Generally, under abiotic stress conditions, sugars function as osmoprotectants and also stabilize the membranes (Afzal et al., 2021). Accumulation of soluble sugars enhances plant survival or tolerance to abiotic stresses, such as drought, heat, chilling and salinity (Mohammadkhani and Heidari, 2008; Sami et al., 2016). Unlike sugars, chlorophylls are the main pigments involved in the process of photosynthesis. The change of chlorophyll content during environmental stresses is a highly visible indicator for quite a few stress events. The ability to maintain chlorophyll content may vary with the genotype, and stress duration and intensity (Monteoliva et al., 2021). Plants that maintain a relatively higher level of chlorophyll under abiotic stresses, such as drought, may directly cause higher photosynthesis rate and yield; and then the higher chlorophyll content would be correlated with stress tolerance (Monteoliva et al., 2021). In the present study, although CIBI treatment did not seem to cause significant change of soluble sugar content in irradiated offsprings, the treatment resulted in obvious changes in chlorophyll content, and a certain proportion of putative chlorophyll-related mutants were generated. Such putative mutants might be used as valuable germplasm materials in *E. ophiuroides* breeding for stress tolerance.

Molecular markers are widely used in plant breeding and have presented advantages in variety and cultivar identification. Owing to their abundance, high rate of polymorphism, ubiquitous distribution throughout the genome, high extent of allelic diversity, and ease of assay by PCR, SSR markers has been widely used to identify radiation-derived variations in DNA sequence in various garden plants and crops (Deng et al., 2022; Ramchander et al., 2022; Taheri et al., 2014). All the polymorphism detected in these studies could be due to irradiation caused insertions and deletions (indels) in DNA sequence, which are highly likely to result in protein reading frame and transcription alteration (Bradshaw, 2016). In the current study, DNA alterations, including increase or decrease in the number of the amplified bands and changes in the size of the amplified fragments, were detected in CIBI-derived mutants of *E. ophiuroides* based on SSR markers. Such changes in DNA sequence might be the direct cause for the occurrences of intragenic or point mutations within a gene and intergenic or structural mutations within chromosomes (Oladosu et al., 2016). Overall, CIBI strategy, being one of the most favored mutagenesis techniques, is an effective and promising way to create and enlarge the mutation materials in grass plants.

## 5 Conclusions

Broadening the germplasm resources of plants in multiple ways is crucial for plant breeding practice due to the bottleneck caused by the limited availability of genetic resources. The study investigated the optimal radiation dose range for CIBI of *E. ophiuroides* seeds and the morphological and physiological responses of *E. ophiuroides* to different doses of CIBI. The results showed that the optimal radiation dose range for CIBI of *E. ophiuroides* seeds was within 50 to 100Gy, and the survival rate of irradiated seeds was 0% when the irradiation dose reached 300Gy. Extensive morphological variability was observed in *E. ophiuroides* under different doses of CIBI. Consequently, the preliminary identification of mutant populations by SSR molecular markers has obtained a number of *E. ophiuroides* mutants, which provides a rich material base for *E. ophiuroides* genetic breeding research. However, the development of plant molecular breeding towards high efficiency and precision has led to the emergence of high-throughput sequencing identification technology as the future trend for mutant screening.

## Data Availability

The original contributions presented in the study are included in the article/**Supplementary Material**, further inquiries can be directed to the corresponding author/s.
