# Supplementary material for: Biological responses of an elite centipedegrass [Eremochloa ophiuroides (Munro) Hack.] cultivar (Ganbei) to carbon ion beam irradiation
Source: Front Plant Sci. 2024 Sep 18;15:1433121. doi: 10.3389/fpls.2024.1433121 (PMC11445083; doi:10.3389/fpls.2024.1433121)
Supplement: Supplementary file 1 [file DataSheet1.docx]

Biological responses of an elite centipedegrass [*Eremochloa ophiuroides* (Munro) Hack.] cultivar (Ganbei) to Carbon Ion Beam Irradiation

**Supplementary data**

^
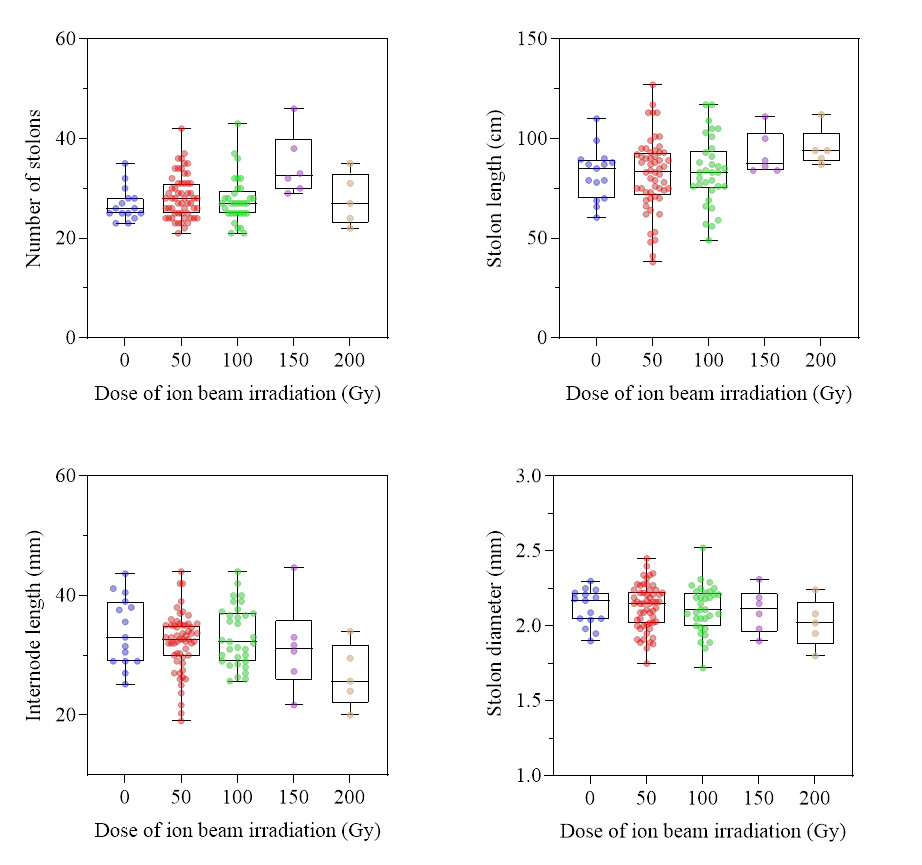
^

**Figure S1**. Effect of CIBI on stolon occurrence and growth characteristics in *E. ophiuroides* during the 2nd growing season. (A) Number of stolons generated. (B) Stolon length. (C) Internode length. (D) Stolon diameter. The first three stolons emerging from a plant were used for determining stolon length, and the length of 3-month-old stolon was measured with a tape measure from the base to tip of the stolon. Internode length and stolon diameter were measured using a ruler or vernier caliper at the position between the third and fourth nodes of the stolon, and five replicates at least were implemented for each index measurement.


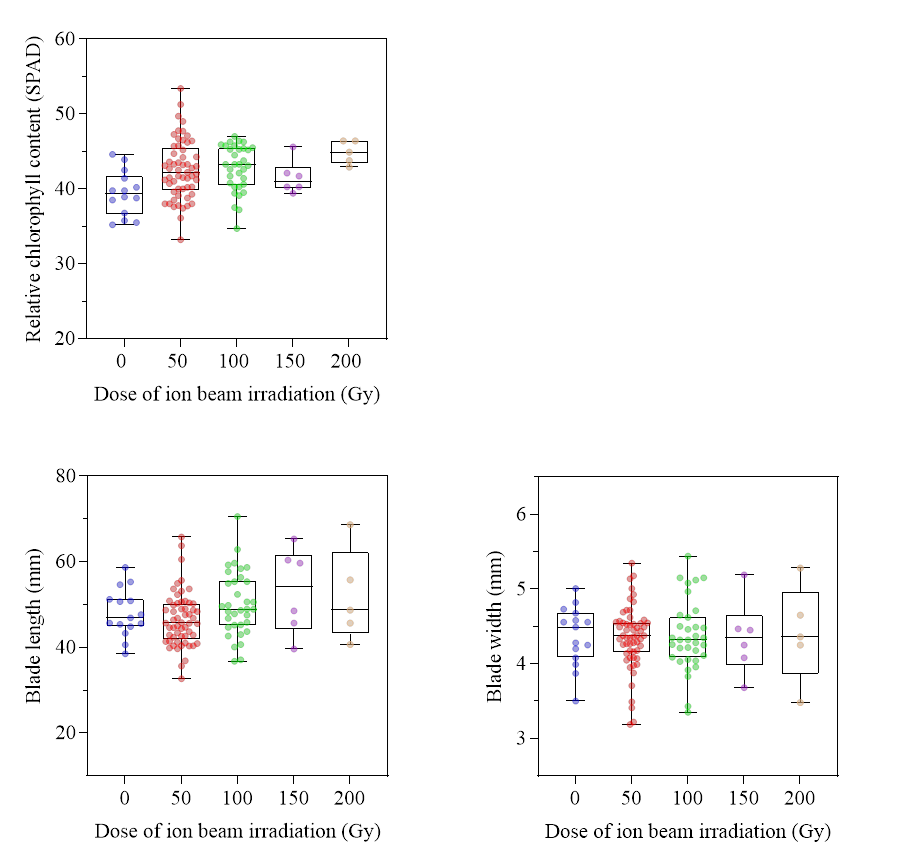


**Figure S2**. Effect of CIBI on chlorophyll content and morphological characteristics of leaf blades in *E. ophiuroides* during the 2nd growing season. (A) Relative chlorophyll content. (B) Blade length. (C) Blade width. The 3rd leaf from tip was used for measuring SPAD value, blade length and width. 10 leaves were taken for each index measurement.

^
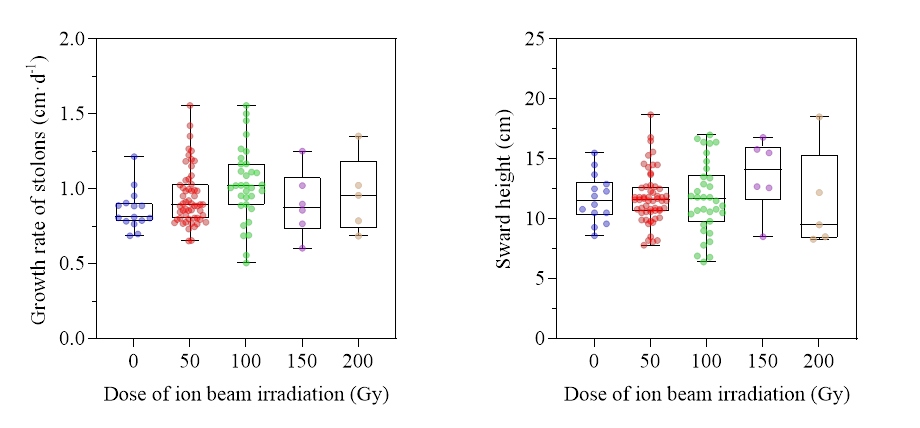
^

**Figure S3**. Effect of CIBI on production potential of *E. ophiuroides* during the 2nd growing season. (A) Growth rate of stolons. (B) Sward height. Growth rate was determined by calculating the elongation of a stolon per unit of growing time with at least five replicates for each plant. Sward height was measured at five different spots from the base level to the highest point of the lawn.

^
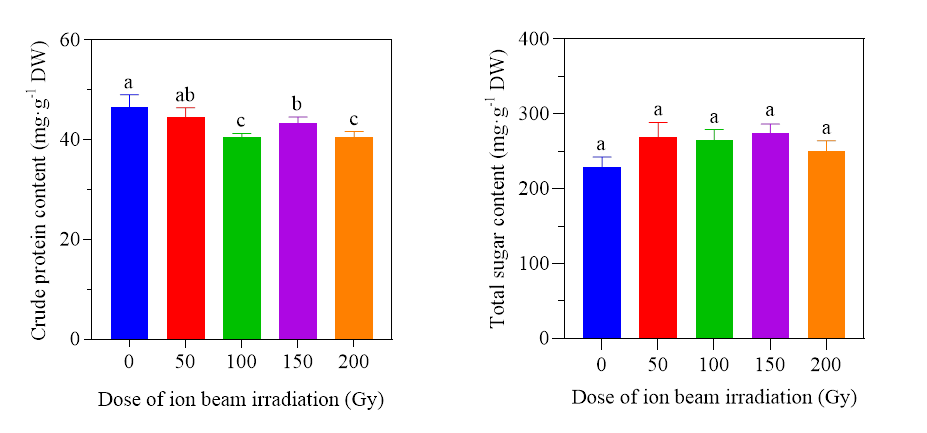
^

**Figure S4.** Effect of CIBI on crude protein content and total sugar content of *E. ophiuroides* during the 2nd growing season. Means with different letters are significantly different between treatments by the Tukey’s HSD test (p＜0.05). (A) Crude protein content. (B) Total sugar content. Five samples were randomly selected from the collected above tissues for both crude protein and total sugar assessment.
